# HIV diagnosis during acute infection: implications of long-acting preexposure prophylaxis and other evolving challenges

**DOI:** 10.1097/COH.0000000000000919

**Published:** 2025-02-17

**Authors:** Tamara Elliott, Daniel Bradshaw, Sarah Fidler

**Affiliations:** a Imperial College London, department of infectious disease, Faculty of Medicine; b Imperial College NIHR BRC; c Imperial College NHS Trust; d UK Health Security Agency, UK

**Keywords:** HIV preexposure prophylaxis, HIV prevention, HIV testing

## Abstract

**Purpose of review:**

Tests for HIV may perform differently in some circumstances such as with preexposure prophylaxis (PrEP) or other HIV prevention agents. Testing algorithms may not account for this, with a risk of false negative or positive HIV results. In this review we have explored the challenges of HIV testing in these special circumstances.

**Recent findings:**

Long-acting injectable PrEP using cabotegravir or lenacapavir has been studied in large randomized controlled trials (HPTN083/084 and PURPOSE1/2 respectively). Injectable PrEP was significantly more efficacious than oral PrEP, but infections still occurred risking the emergence of HIV drug-resistance. HIV diagnostic test results were atypical in those receiving injectable PrEP, with low or undetectable HIV viral loads, delayed or diminished antibody, and HIV detection assays reverting from reactive to unreactive; so-called long acting early viral inhibition (LEVI) syndrome. In these cases, missed or delayed HIV diagnoses could be reduced with the use of HIV nucleic acid amplification tests in addition to routine testing, but this remains unfeasible in many settings.

**Summary:**

Finding HIV testing strategies that are affordable and practical in low- and middle-income countries that can accurately diagnose HIV in the context of HIV prevention is of high importance, but more research is needed in this area.

## INTRODUCTION

In 2023, there were 1.3 million new HIV diagnoses globally, almost half of whom were cisgendered women and girls [1]. Preexposure prophylaxis (PrEP) has been initiated in over 7 million people since it became available in 2012 [2]. Globally, UNAIDS highlighted that despite increases in PrEP use, this falls short of the target of 21 million by 2025 [3], and substantial inequalities are observed for PrEP use in different populations affected by HIV [4].

Currently, oral PrEP as a combination of tenofovir disoproxil (TDF) or tenofovir alafenamide (TAF) with emtricitabine (FTC), is recommended and available in many settings, but other antiretroviral delivery modalities have become available. In sub-Saharan Africa, the dapivirine vaginal ring (DVR) is available for women [5], and long-acting injectable PrEP has demonstrated excellent results in clinical trials. An extended-release formulation of cabotegravir received US Food and Drug Administration (FDA) approval for PrEP in 2021 [6]. For people who require PrEP it is important to be able to reliably test for HIV so that if HIV is acquired, PrEP regimens can be switched to appropriate antiretroviral therapy (ART) to avoid the development of ART resistance [7]. In a study looking at the effects of PrEP in New York City, for those with a new diagnosis of HIV, recent PrEP use was associated with a 7 times greater risk of M184I/V mutations compared to those with no known use [8^▪^]. In the context of PrEP, HIV testing and diagnosis can become more complicated. This carries not only medical and logistical challenges, but is also a source of extreme anxiety and uncertainty to those affected [9–11]. For those who may benefit from access to PrEP but do not identify as such due to societal, structural, personal, stigma related, or legal reasons [12], accessing PrEP services is further challenged by potential inaccuracies in HIV testing.

Throughout this article we will explore circumstances in which there may be challenges in confirming HIV status. We will review different HIV testing algorithms and look at the strategies used to address these challenges. 

**Box 1:**
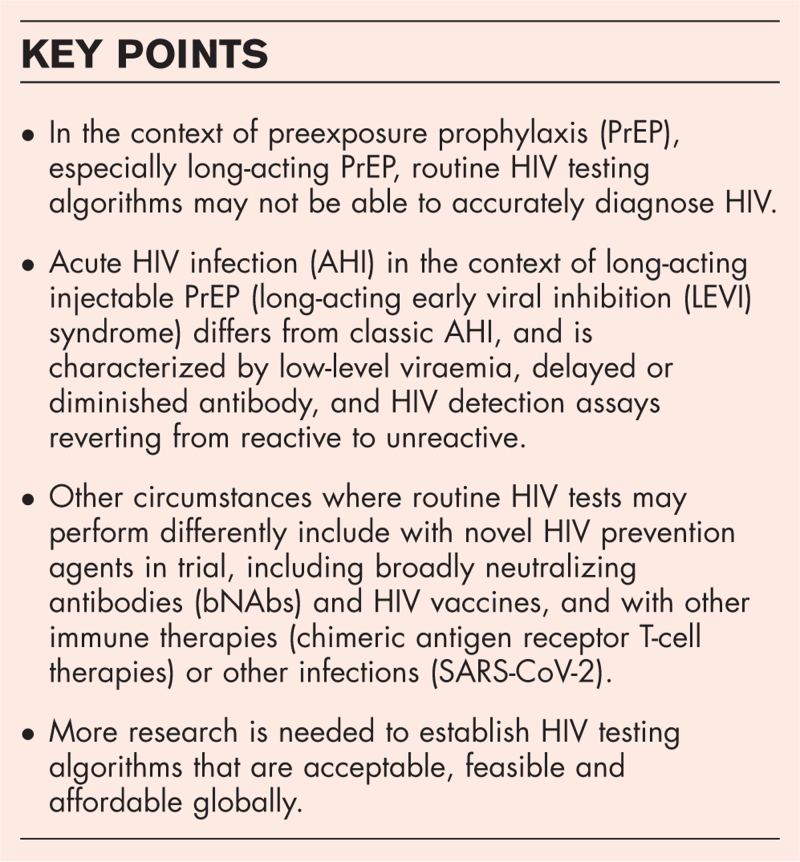
no caption available

## HIV TESTING

Tests for HIV detect specific antibody, antigen or genetic material. The ability for an assay to detect HIV depends on the target tested and the timing in relation to acquisition [13]. In acute HIV infection (AHI), the earliest testable marker is HIV RNA, followed by the transient HIV capsid protein p24. Antibodies [immunoglobulin M (IgM) then immunoglobulin G (IgG)] become detectable later in infection (with a range of 8–90 days after first PCR positivity) [14–16]. To capture AHI, and shorten the window period from HIV acquisition to diagnosis, most testing algorithms recommend antigen-antibody (Ag/Ab) tests which detect both IgM/IgG and p24 antigen. The addition of nucleic acid amplification testing (NAAT) for detection of HIV genetic material may be required for very early infection. However, these tests often require specialized transport systems, laboratory infrastructure and technical expertise which are costly to establish and maintain, and may not be widely available in many low- and middle-income countries (LMICs). HIV rapid or point-of-care tests (POCTs) are widely available globally, and can be used for HIV self-testing (both blood and oral fluid) [17,18]. Although some POCTs detect p24 antigen as well as antibody, most detect antibody only (either IgM with IgG or IgG only) and these are therefore unsuitable to detect AHI. HIV NAATs for near-patient testing are now also available [19,20], although unlike other POCTs still require specialized equipment and are not suitable for HIV self-testing. A key population for which these have been used in LMICs are HIV-exposed infants where false positive serological tests due to maternal antibodies are replaced by NAAT testing. Other assays that are not used routinely but may aid diagnosis include western blot and line immunoassays. These detect HIV specific antibodies to viral proteins but have a longer processing time and higher cost [21]. In circumstances where additional testing is required, HIV proviral DNA from whole blood or peripheral blood mononuclear cells can also be used.

## LONG-ACTING INJECTABLE PREEXPOSURE PROPHYLAXIS

**FIGURE 1:**
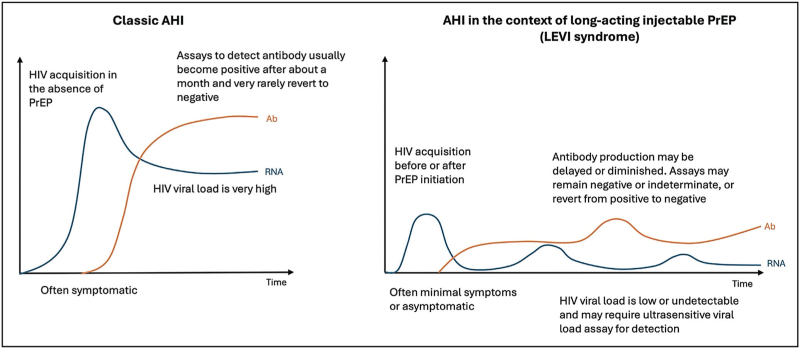
A simplified illustration of the differences between classic acute HIV infection and long-acting early viral inhibition (LEVI) syndrome. The first panel to the left demonstrates the features of classic acute HIV infection (AHI) where there is a rapid increase in HIV viral load which then declines to a set point. HIV specific antibodies become detectable later in infection after a reduction of viraemia. In AHI in the context of long-acting injectable PrEP (LEVI syndrome, right panel) [26^▪▪^] the HIV viral load may be low or undetectable, and the detection of HIV specific antibodies may be delayed or diminished, and revert from positive to negative. Ab, antibody; AHI, acute HIV infection; LEVI, long-acting early viral inhibition; PrEP, preexposure prophylaxis.

The use of cabotegravir long-acting PrEP given 8-weekly was studied in two large randomised controlled trials in cisgender men and transgender women (HPTN083 [22]) and in cisgender women (HPTN084 [23]). In these studies, injectable PrEP far outperformed oral formulations, leading to the US FDA to approve cabotegravir PrEP. However, there were still cases of HIV acquisition on cabotegravir both during the blinded portion of the trial and the open-label extension [24^▪^,^25^]. Between both HPTN083 and HPTN084 there have been 41 HIV diagnoses amongst 3446 participants randomised to cabotegravir PrEP [26^▪▪^]. The HIV tests used in these clinical trials are summarized in Table 1. HIV diagnosis was delayed in 17 of these cases when an FDA approved HIV POCT and Ag/Ab test were used for screening. Often HIV-1 RNA was low or undetectable and antibody expression was delayed or diminished. Additionally, HIV-1 DNA levels were low or undetectable in those on cabotegravir versus oral PrEP [27]. Reports of HIV detection assays (including HIV RNA, Ag/Ab, and confirmatory Ab tests) reverting from positive/reactive to negative/unreactive were also seen [28]. The altered presentation of AHI in those receiving cabotegravir PrEP led the investigators to term this “long acting early viral inhibition” (LEVI) syndrome (Fig. 1) [26^▪▪^,^29^]. Emergence of integrase strand transfer inhibitor (INSTI) resistance also occurred in recipients of cabotegravir PrEP [25,28], and was seen in 10 of 14 participants that had a delayed diagnosis and received cabotegravir PrEP within 6 months of a positive HIV test [26^▪▪^]. The use of HIV RNA testing was found to better detect HIV in the context of cabotegravir PrEP, and therefore reduce the risk of INSTI resistance [30]. However, since HIV RNA levels are often low or undetectable in the context of long-acting cabotegravir PrEP, there remains a possibility of false negative results. HPTN083/084 investigators are now evaluating the use of RNA testing to screen for HIV in those on cabotegravir PrEP in the open label extensions of these studies [26^▪▪^].

**Table 1:** HIV testing in long-acting injectable PrEP trials

	HPTN 083/084 [23,28]	PURPOSE 1/2 [34^▪▪^,^35^^▪▪^]
Study enrolment/initiation	• HIV RNA test within 14 days prior to enrolment^a^ • 1 or 2 FDA approved rapid HIV tests^a^ • Lab Ag/Ab test^a^	• FDA approved rapid Ag/Ab test^7^ • Lab Ag/Ab test^8^ • HIV RNA test (quantitative)^9^
Routine study visit	• 1 or 2 FDA approved rapid HIV tests^a^ • Lab Ag/Ab test^a^	• Rapid Ag/Ab test^7^ • Lab Ag/Ab test^8^
Additional tests in cases of an indeterminate, reactive or positive result	• Lab Ag/Ab^1^ • Confirmatory Ab test^2^ • HIV RNA test (qualitative)^3^ • HIV RNA test (quantitative)^4^ • Single copy RNA test^5^ • Ultra-sensitive HIV DNA^5^ • HIV resistance testing^6^	• Confirmatory Ab test^2^ • HIV RNA test (quantitative)^9^ • HIV RNA test (qualitative)^10^ • HIV recency assay^11^ • HIV resistance testing

a Locally available assays: ^1^Architect HIV Ag/Ab Combo assay, ^2^Geenius HIV 1/2 Supplemental assay, ^3^Aptima HIV-1 RNA Qualitative Assay, ^4^Abbott RealTime HIV-1 Viral Load Assay, ^5^laboratory developed assay, ^6^GenoSure PRIme, PhenoSense INSTI with cabotegravir, ^7^Determine, Abbott, ^8^Siemens, ^9^Cobas 6800, ^10^Cobas Ampliprep-cobas TaqMan 2.0, ^11^Lag-EIA, Sedia Biosciences.

HIV RNA testing is not feasible or affordable in many settings where cabotegravir long-acting PrEP is available. The use of HIV NAAT near-patient POCTs may represent an option where available but is still likely to be more expensive than antibody testing. Further, the limit of detection is higher than for most laboratory-based assays (800 c/ml for HIV-1 RNA using EDTA plasma for m-PIMA [20]; and 278 c/ml or 668 c/ml for total HIV-1 nucleic acids in whole blood or dried blood spot (DBS) respectively using the Xpert HIV-1 Qual test [31]). Such tests could therefore miss low RNA levels which have been described in LEVI. In an analysis from HPTN083, comparable performance was seen for the Xpert HIV-1 viral load test [19], which used plasma, versus reference assays. However, the Xpert HIV-1 Qual test, which used DBS, was less sensitive [32].

In an open-label extension of HPTN083, including 2620 individuals receiving cabotegravir PrEP, 29 acquired HIV; 5/29 (17%) were diagnosed after an isolated positive HIV RNA, with the remaining 24/29 (83%) diagnosed by multiple positive tests [33]. However, 23 other individuals had an isolated positive HIV RNA of whom 22/23 (96%) were later determined to be HIV negative by repeat testing, and 1/23 remained of indeterminate status. Median HIV viral load in individuals with false positive results were significantly lower than true positives (below the limit of quantification versus 1597 c/ml, respectively). The positive predictive value for detecting HIV by RNA screening for participants with cabotegravir PrEP in the past 6 months was only 9.1% versus 60% without [33].

Although not yet available outside of a trial setting, twice yearly injectable lenacapavir (a first-in-class capsid inhibitor) has demonstrated outstanding results for the prevention of HIV. Results from PURPOSE1, a large randomised controlled trial in cisgender women in South Africa and Uganda, showed zero new HIV infections in 2134 taking lenacapacvir PrEP, compared to 55 of 3204 taking oral PrEP over the same time period [34^▪▪^]. Data from PURPOSE2, which included cisgender men, and transgender and nonbinary people demonstrated just 2 new HIV infections in 2179 participants taking lenacapavir PrEP compared to 9 cases out of 1087 taking oral PrEP [35^▪▪^]. Both participants with an HIV diagnosis on lenacapavir PrEP were asymptomatic, and retrospective testing with standard HIV-1 RNA tests did not reveal delayed diagnosis for either participant. However one participant did have an isolated positive HIV-1 RNA single copy test on retrospective testing at the visit prior to diagnosis, and both had the N74D capsid resistance mutation at diagnosis [35^▪▪^]. The impressive efficacy of lenacapvir PrEP was particularly evident in cisgendered women – this is important as oral PrEP has traditionally been shown to be less efficacious in women [36–39], who make up almost half of new infections. This is primarily due to issues with uptake and adherence [40] so access to injectable PrEP, and in particular lenacapavir PrEP which is only administered twice yearly, is a priority in this group. In recognition of the importance of access of lenacapavir PrEP to populations in need, Gilead have announced it will licence generic lenacapavir in 120 LMICs [41].

In 2022, the World Health Organization (WHO) included long-acting cabotegravir in its recommendation for PrEP [42]. INSTIs are widely used for ART, and dolutegravir (DTG) is recommended by the WHO as a first- and second-line treatment. However, resistance to DTG has been increasing with up to 20% of those on DTG who are not virally suppressed showing evidence of resistance [43,44]. An increase in INSTI-resistant HIV in the population could have consequences for those starting cabotegravir PrEP, with the likelihood of reduced efficacy, as well as implications for treatment options for those that acquire INSTI-resistant HIV. In high income countries, HIV drug resistance testing is widely available which allows for tailoring of the ART regimen, but this is not the case in most resource limited settings, and is not recommended by WHO [44].

With the continued roll out of cabotegravir PrEP and hopefully lenacapavir PrEP, practitioners need to be vigilant for AHI, with an awareness that routine testing may not be sufficient. With more sophisticated testing unavailable in many settings, diagnosis of the rare cases of HIV acquisition on injectable PrEP may be delayed leading to a very real possibility of drug resistance.

## CURRENT GUIDELINES FOR HIV TESTING IN ORAL OR INJECTABLE PREEXPOSURE PROPHYLAXIS-USERS

Within different national and international PrEP guidelines, recommendations for HIV testing differ substantially both for oral and injectable formulations (see Table 2).

**Table 2:** Comparison of approaches to HIV testing in oral or injectable PrEP-users within national and international guidelines

		BHIVA/BASHH 2024	CDC 2024	WHO 2024
Oral PrEP initiation	Sample	Serum, plasma, blood	Plasma, blood	Serum, plasma, blood, OF
	POCT	Yes	Yes	Yes
	HIVST	No	No	Yes
	Serology	Ag/Ab^a^, Ab	Ab/Ag^b^, Ab	Ab/Ag, Ab
	RNA if acute HIV suspected	Yes	Yes	No^c^
	Deferral if possible acute HIV	Yes	No^d^	No
Oral PrEP monitoring	Sample	Serum, plasma, blood	Plasma	Serum, plasma, blood, OF
	POCT	Yes	No	Yes
	HIVST	Yes	No	Yes
	Serology	Ab/Ag, Ab	Ab/Ag	Ab/Ag, Ab
	RNA	No	Yes	No
	Frequency	3–6 months	At least 3 months	Usually 2–3 months
	Testing post cessation	At 45 days post end of PrEP	No	No
Injectable CAB PrEP initiation		As for oral PrEP^e^	As for oral PrEP	Need further evidence
Injectable CAB PrEP monitoring	Sample	Serum/plasma	Plasma	Need further evidence
	Tests	Ab/Ag and RNA	Ab/Ag and RNA	Need further evidence
	Frequency	2 months	2 months	Need further evidence
Inconclusive HIV results	Additional tests	Ab/Ag, western blot, RNA, DNA	Ab/Ag, RNA, specialist tests	Ab, Ab/Ag, rarely virological tests
	Specialist advice	Yes	Yes	Not specified
	Management – Oral PrEP	Not specified	Continue/intensify/pause	Continue/pause
	Management – Injectable CAB PrEP	Not specified	Pause	Not specified

Ab/Ag, antibody-antigen, BHIVA/BASHH, British HIV Association/British Association for Sexual Health and HIV, CAB, cabotegravir, CDC, Centers for Disease Control; HIVST, HIV self test, OF, oral fluid, PrEP, HIV pre exposure prophylaxis, POCT, point of care test, WHO, World Health Organisation.

a Lab Ag/Ab required but can start with negative POCT Ab pending result.

b Lab Ag/Ab preferred.

c Repeat serology at 2 weeks.

d As long as POCT negative, pending RNA test.

e Except additional RNA test on day of switch from oral PrEP.

Prior to oral PrEP initiation, the CDC [45] and BHIVA/BASHH (in consultation [46]) recommend blood rather than oral-fluid based testing given concerns about lower sensitivity for oral-fluid. By contrast, WHO recommends either approach is acceptable, likely reflecting its focus on reducing barriers to PrEP initiation in LMICs [18]. Similarly, WHO alone recommends the option of HIV self-testing for oral PrEP or the DVR, and does not advise routine HIV RNA testing where AHI is suspected, rather recommending repeat serology. Monitoring in oral PrEP users also differs, with the CDC recommending 2-monthly HIV Ag/Ab plus RNA; BHIVA/BASHH 3–6 monthly blood-based antibody or Ag/Ab, including HIV self-testing; and WHO usually 2–3 monthly blood or oral-fluid-based antibody or Ag/Ab testing.

For initiation of cabotegravir PrEP, HIV testing recommendations are as for oral PrEP, although BHIVA/BASHH advises baseline HIV-1 RNA in addition to Ag/Ab testing if switching from oral PrEP. For monitoring, both CDC and BHIVA/BASHH recommend 2-monthly Ag/Ab and RNA testing in accordance with the FDA label [47]. The WHO does not recommend HIV self-testing for cabotegravir PrEP, and highlights the need for further data to inform guidance. The WHO also raises concerns for the feasibility of monitoring with HIV RNA in LMICs, citing a systematic review in which the median time to HIV NAAT results was 35 days [48]. However, they state that if there is a suspicion of AHI then a NAAT could be considered if available, but alternatively PrEP can be deferred and antibody tests can be repeated in four weeks [5].

Where indeterminate HIV test results are observed in PrEP-users, BHIVA/BASHH recommend expert regional consultation or referral to a national specialist service. The potential role of HIV-1 proviral DNA testing is highlighted, which may be detectable even when suppression of HIV replication by PrEP has led to an undetectable plasma RNA. The CDC similarly recommends expert consultation and specialized testing in this group and advises three management options: continuing, intensifying or pausing PrEP for 1–2 weeks whilst repeat samples are collected. Intensification is particularly advised in individuals who may have missed doses. The rationale for pausing PrEP in some individuals is to allow viral replication and thereby increase the likelihood of detection by NAAT, but potentially exposes an HIV-negative person to HIV, so alternative protection should be offered whilst off PrEP. For cabotegravir PrEP users, the CDC recommends pausing injections for 1–2 weeks with repeat sampling until HIV infection is excluded. The WHO recommends repeating serology after 2 weeks whilst continuing PrEP and if the HIV status remains inconclusive, to consider pausing PrEP for up to 4 weeks, and perform both serological and virological tests at 2 weeks. Serological tests alone could prove problematic due to the delays in antibody detection seen in the cabotegravir PrEP clinical trials [28], and the tail-phase of cabotegravir following cessation [49].

## BROADLY NEUTRALIZING ANTIBODIES FOR HIV PREVENTION

Passive infusions with HIV-specific broadly neutralizing antibodies (bNAbs) have the potential to induce protection against HIV [50]. Preclinical trials using bNAbs in nonhuman primates (NHP) have resulted in protection from HIV following challenge experiments [51–53]. However, translation to humans remains challenging [54], and at the time of writing, no clinical trials in humans have successfully recreated the efficacy outcomes reported in NHP challenge models.

Several bNAbs have been assessed as monotherapy [55] and combined therapy [56,57] in phase 1 clinical trials, but only VRC01 (a bNAb targeting the CD4-binding site) has advanced to phase 2 in the antibody mediated prevention (AMP) trials [58]. Although it was not efficacious overall, for those exposed to bNAb sensitive virus it was up to 75% protective [59] and serum concentrations of VRC01 correlated with protection [60]. HIV testing in the context of bNAb prevention trials is complex. It is possible that passive infusion of HIV-specific bNAbs may result in false positive antibody tests. However, in the VRC01 trial, human sera containing purified VRC01 up to 1600 mcg/ml did not induce any reactivity in commercially available HIV test kits [58]. In the AMP trials four-weekly Ag/Ab assays were used for initial HIV testing, with additional HIV RNA testing for an inconclusive or reactive result. A blood redraw for confirmatory testing was taken, and if necessary tests for HIV-1 DNA were performed [58].

## HIV PREVENTIVE VACCINE TRIALS

To date none of the recent HIV prevention vaccine trials have demonstrated efficacy although most do induce the production of HIV-specific antibodies detectable with standard HIV-antibody tests [61–63], and so most HIV vaccine trials use HIV RNA and/or DNA testing to explore primary efficacy outcomes. Vaccine-induced seropositivity (VISP) varies depending on the constructs used, and may be long-lasting, in some cases up to 20 years after vaccination [64]. For most people with VISP, HIV rapid detection tests will result in false positive results if no other tests are used [62,65]. For people who have previously enrolled into such studies, additional tests may be required to determine final HIV status. If an effective HIV vaccine were to be developed and rolled out more widely, this could have implications for HIV testing programmes globally, perhaps requiring the addition of HIV NAAT which may be problematic in many settings.

## SARS-CoV-2

Recent SARS-CoV-2 infection may present challenges for HIV serology interpretation. In one study, detection of SARS-CoV-2 RNA within two weeks of a laboratory HIV Ag/Ab test was associated with a false positive HIV result [66]. The authors speculated this could relate to HIV and SARS-CoV-2 antigenic cross-reactivity, for example due to short regions of viral genome and/or structural protein similarity [66]. Another recent study found an association between SARS-CoV-2 spike IgG positivity in people with prior history of COVID-19 and a false positive laboratory HIV Ag/Ab test, with an HIV false positive proportion of 1.8% versus 0.4% as predicted by CDC analyses [67].

## CHIMERIC ANTIGEN RECEPTOR T-CELL THERAPIES

The expansion in use of chimeric antigen receptor T-cell therapies (CAR-T) for haematological cancers may also pose challenges for HIV diagnosis. The therapy involves use of a modified, replication-incompetent, self-inactivating form of HIV-1 which integrates into host DNA to induce long-term transgene expression of chimeric antigen receptors by T-cells, enabling them to destroy specific tumour cells. Gene products expressed through this lentiviral-vector based technology may include HIV-1 Long Terminal Repeat or Gag, resulting in false positive HIV-1 NAAT in the context of negative Ag/Ab testing [68].

## CONCLUSION

Throughout this review we have explored scenarios where HIV diagnostics may be challenging. The increasing availability of long-acting PrEP has the potential to dramatically alter the HIV epidemic, but the ability to accurately diagnose HIV at initiation and maintenance is essential. However, we have seen from trial data that diagnoses can be missed or delayed, potentially resulting in the development of HIV drug resistance. Regular HIV RNA testing can reduce this risk but is not universally available and so not recommended in many LMIC settings. Additionally, in the long-acting PrEP trials, some HIV diagnoses were missed prior to enrolment, so enhanced HIV testing prior to initiation might be required particularly for those with symptoms of AHI. Uncertainty of HIV status confers an enormous emotional and practical burden to PrEP providers and users, and better engagement with populations with high HIV incidence as well as healthcare providers may aid understanding of the HIV testing landscape. Finding testing strategies that are acceptable, feasible, affordable and practical globally is key, and more data is needed in this area.

## Acknowledgements

*None.*

### Financial support and sponsorship

*T.E. and S.F. are supported by the NIHR Imperial Biomedical Research Centre (BRC).*

### Conflicts of interest

*There are no conflicts of interest.*

## REFERENCES AND RECOMMENDED READING

Papers of particular interest, published within the annual period of review, have been highlighted as:

▪ of special interest
▪▪ of outstanding interest
